# Complex Effects of a Land-Use Gradient on Pollinators and Natural Enemies: Natural Habitats Mitigate the Effects of Aphid Infestation on Pollination Services

**DOI:** 10.3390/insects14110872

**Published:** 2023-11-13

**Authors:** Tal Shapira, Tohar Roth, Adi Bar, Moshe Coll, Yael Mandelik

**Affiliations:** 1 Department of Entomology, Faculty of Agriculture, Food and Environment, The Hebrew University of Jerusalem, Rehovot 7610001, Israel; tal.shapira@mail.huji.ac.il (T.S.); tohar.roth@mail.huji.ac.il (T.R.); moshe.coll@mail.huji.ac.il (M.C.); 2 The Advanced School for Environmental Studies, The Hebrew University of Jerusalem, Rehovot 7612001, Israel

**Keywords:** agroecology, ecosystem services, herbivores, land-use, Mediterranean agro-ecosystem, parasitoid wasps, pests, wild bees

## Abstract

**Simple Summary:**

Pollination and biological pest control by insects are crucial ecosystem services for agriculture. The activity of pollinators and natural enemies (natural pest control providers), is influenced by land-use and by interactions among these organisms. However, little is known about the combined effects of such factors on the activity of these beneficial organisms and on their ultimate effects on crop yield. We studied how the characteristics of natural habitats, nearby vegetation and the presence of herbivorous pests affect pollination, natural pest control and seed set by model plants. The study was conducted in a Mediterranean agro-ecosystem, where we placed caged and uncaged potted model plants that were either aphid-infested or aphid-free. We quantified the activity of pollinators on model plant flowers, aphid predation and parasitism by natural enemies, as well as fruit and seed set. Our findings revealed a notably stronger positive effect of natural areas on pollinator activity when plants were infested with aphids compared to aphid-free plants. This suggests a potentially crucial role of natural habitats in lessening the negative impact of aphid infestation on pollination services. These results highlight the complex effects of land-use on pollinators, natural enemies and plant productivity.

**Abstract:**

Pollinators and natural enemies are essential ecosystem service providers influenced by land-use and by interactions between them. However, the understanding of the combined impacts of these factors on pollinator and natural enemy activities and their ultimate effects on plant productivity remains limited. We investigated the effects of local and landscape vegetation characteristics and the presence of herbivorous pests on pollination and biological control services and their combined influence on phytometer seed set. The study was conducted in a Mediterranean agro-ecosystem, encompassing ten shrubland plots spanning a land-use gradient. Within each plot, we placed caged and uncaged potted phytometer plants that were either aphid-infested or aphid-free. We quantified insect flower visitation, aphid predation and parasitism rates, and fruit and seed set. We found scale-dependent responses of pollinators and natural enemies to land-use characteristics. Flower species richness had a positive impact on aphid parasitism rates but a negative effect on pollinator activity. Notably, we found a more pronounced positive effect of natural areas on pollinator activity in aphid-infested compared to aphid-free plants, indicating a potentially critical role of natural habitats in mitigating the adverse effects of aphid infestation on pollination services. These results highlight the complex and interactive effects of land-use on pollinators and natural enemies, with significant implications for plant productivity.

## 1. Introduction 

Ecosystem services like pollination and biological pest control are crucial for agriculture [1]. The presence of diverse and abundant pollinator and natural enemy communities positively influences the delivery of these services [2]. To support these communities, especially bees [3] and arthropod predators and parasitoids [4], extensive efforts focus on land-use planning and field management that provide necessary resources for these guilds in agro-ecosystems [5,6]. However, studies traditionally explored the effects on either pollinators or natural enemies, with limited attention on their interactive responses. However, recognizing that these groups may influence each other is essential, as such interactions would significantly impact their effectiveness in providing ecosystem services [7]. Therefore, there is a growing interest in understanding these interactions under real-world field conditions and their effects on plant production and crop yield, although studies on this topic remain limited [8]. 

The interactions between co-occurring pollinators and natural enemies can result in positive, negative, or neutral effects on ecosystem service delivery depending on underlying mechanisms [8,9]. Positive (synergistic) interactions may boost plant productivity, with natural enemies indirectly benefiting pollinators by controlling pests, contributing to increased visit rewards and availability of flowers, especially when dealing with florivorous pests [10,11]. Greater pollinator activity can, in turn, enhance floral diversity, benefiting natural enemies [12]. Negative (antagonist) interactions indicate reduced effectiveness of pollinators when pest control activity is high, or vice versa, and often occur due to resource competition (e.g., [13]). Some studies report the decreased abundance of parasitoid [14] or predatory hoverfly [15] in the presence of high bee activity, while others note reduced flower visitation by bees in the presence of generalist predators, such as predatory bugs [16] and ants (e.g., [17]). Neutral interaction effects may imply independence (additive relationship) between guilds or that only one guild primarily contributes to plant productivity [18].

Ecosystem service provisioning is influenced not only by inter-guild interactions but also by landscape and habitat characteristics, as demonstrated in several studies [2,19,20,21,22,23,24,25]. For instance, Dainese et al. [2] conducted a comprehensive meta-analysis revealing that landscape simplification significantly reduced the richness and abundance of both pollinators and natural enemies. However, it is important to note that other meta-analyses have reported varying and sometimes inconsistent responses of these guilds to landscape composition [19,20,22]. Nonetheless, it is widely observed that semi-natural habitats generally play a crucial role in enhancing pest control and pollination services [21]. Furthermore, the specific type of semi-natural habitat can exert varying influences, both at local and landscape scales [23,25]. Lastly, local habitat elements, such as flower strips and hedgerows, have also been shown to impact pollinators and natural enemies, although the effects can vary [24]. Proximity to flower strips has been found to enhance both pollination and pest control services, whereas greater flowering plant diversity within these strips seems to improve only pollination services [24]. 

It seems therefore that research often focuses on the response of individual guilds to landscape and habitat factors separately, overlooking potential combined effects and interactions [7,26]. A comprehensive approach is necessary to understand the mechanisms behind pollination and pest control service delivery and to develop integrated pest and pollinator management strategies [7,26]. Nonetheless, studying multi-guild interactions across diverse landscapes and habitats remains challenging [8], including the integration of data collected through various methods that are suitable for studying different organisms within the system. In our study, conducted in a Mediterranean agro-ecosystem along a land-use gradient, we used phytometer plants to simultaneously observe pollinator activity, aphid predation, parasitism rates, and fruit and seed set. Our research addressed two key questions:

(1) What are the combined and interactive effects of pollinators, pests, and their natural enemies on fruit and seed set in an agro-ecosystem?
(2) How do these interactions vary with local and landscape habitat characteristics?

## 2. Methods

### 2.1. Study Area

The research was conducted in the Judean Foothills, an agro-ecosystem located in central Israel (31.6–31.9° N, 34.7–35.0° E, with elevations ranging from 60 to 280 m above sea level). This region is characterized by a diverse landscape comprising annual crop fields, orchards, and various natural and semi-natural habitats, including herbaceous areas, shrublands, and planted forests. Situated on the boundary between a humid Mediterranean ecosystem to the north and an arid ecosystem to the south, this area is a biodiversity hotspot, particularly of bees [27] and plants [28]. Notably, honeybees (*Apis mellifera*) are commonly managed in this region for crop pollination and honey production, while wild and feral honeybees are absent due to infestations of parasitic *Varroa* mites.

### 2.2. Experimental Design

**Figure 1:**
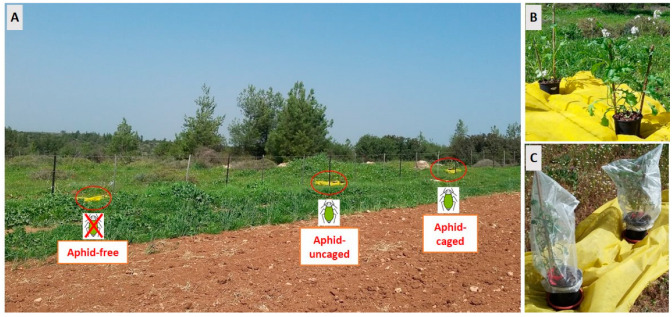
(**A**) Overview of the experimental study plot depicting the three distinct aphid treatments, the surrounding landscape, and the neighboring crop-free field. (**B**) Close-up view of the “Aphid-uncaged” treatment, showing plants infested with aphids and left exposed. (**C**) Close-up view of the “Aphid-caged” treatment, showing plants infested with aphids and covered with dense organza bags to prevent insect access.

Ten study plots, 400 m^2^ each, were established within natural habitats bordering agricultural fields that were devoid of crops during the study period (i.e., prior to sowing). The inclusion of adjacent crop-free fields enabled us to minimize potential variations arising from crop-specific effects. Plot selection aimed to create a gradient of landscape composition, based on the ratio between natural/semi-natural habitats and agriculture within a 1 km radius of each plot. The proportion of natural/semi-natural habitats within this radius ranged from 10% to 70% (see Figure S1). Plot centers were at least 500 m apart and distributed across three geographic sub-regions. Within each plot, we placed three pairs of potted blooming greenhouse-grown *Diplotaxis erucoides* plants, which served as phytometers, assigned to three treatments (further details on treatment application are provided below): (1) Aphid-infested (*Myzus persicae*) uncaged plants—allowing insects unrestricted access to the plants (“aphid-uncaged” treatment). (2) Aphid-infested caged plants—protecting plants with dense organza bags to prevent insect access (“aphid-caged” treatment). (3) Aphid-free plants with open access to insects (“aphid-free” treatment). Infested phytometer plants were kept caged during transport to the field to prevent aphid movement among plants. The plants were positioned within natural habitat patches, 10 m away from the patch edges to minimize edge effects. Each pair of plants (i.e., same treatment) was spaced 10 m apart from each other (see Figure 1) and placed on a 1 m^2^ yellow cloth to enhance visibility to nearby flying insects (as earlier studies in this system yielded very low insect activity on small plant patches). The small size of the cloth and the uneven micro-topography of the landscape limited the cloth visibility to short distances. Furthermore, the cloth was applied equally to all treatments in the experiment and is implausible to have interacted with treatment effects. Potted plants were watered daily and left in the field for five days.

### 2.3. Phytometer Species and Aphid Infestation

Our selected phytometer species was *Diplotaxis erucoides* (Brassicaceae), a Mediterranean annual herb, known for its self-incompatibility, reliance on pollinators, and prevalence in our study region. Sixty potted *D. erucoides* were grown for seven weeks from seeds in a greenhouse at 20 °C. During that time, flower buds were removed to maximize vegetative growth. When plants were ca. 40 cm high, we ceased removing buds and allowed the plants to flower for another week. Ten to 14 days prior to moving these plants to the field, two-thirds of them were isolated in the greenhouse and then infested with 20 adults *Myzus persicae* (Hemiptera: Aphididae) each that were placed on two leaves per plant, using a fine camel-hair brush. *M. persicae* is a highly polyphagous herbivore and a major pest of a wide range of cruciferous, cucurbits, solanaceous, and malvaceous crops worldwide, as well as various ornamental and weedy plants [29]. The *M. persicae* specimens were obtained from a laboratory-reared colony, which was routinely inspected for (absence of) parasitoids. Upon transfer to the field, the density of aphids on infested phytometer plants ranged between 55 and 150 adult aphids per plant. Plants with similar aphid densities were paired and then randomly assigned to the aphid-infested treatments.

### 2.4. Data Collection

Field work was conducted for one month in 2018, between early March to early April, under standardized and optimal weather conditions; sunny days, 16–34 °C, <2.5 m/s wind velocity (measured a few times during each field working day). We recorded the following parameters:

(a) Landscape and local habitat characteristics

Our analysis of landscape characteristics involved calculating the percentage of natural/semi-natural habitats within varying radii (100, 250, 500, 750, 1000, 1500, and 2000 m) around each study plot using Esri ArcMap, version 10.6.1 (refer to Figure S1). This computation was performed based on the Israel land-use map (Central Bureau of Statistics) and involved ground-level validation. The category of natural/semi-natural habitats encompassed grasslands, shrublands, and planted forests. For local habitat characteristics, we assessed blooming plant species richness and abundance within each plot. This assessment was based on ten samples of randomly placed 1 m diameter hoops across the entire plot, where we thoroughly recorded all blooming plant species and their respective abundance levels (number of flowers/inflorescences), following the methodology detailed in Roth et al. [30]. The plant survey was conducted immediately after the phytometers were transported back to the laboratory.

(b) Pollination services

To assess pollination services provided to phytometers, we recorded pollinator activity and monitored fruit and seed set in the uncaged treatments (both infested and uninfested), where plants were exposed to insect activities. To quantify pollinator activity, we observed phytometer flowers over two consecutive days, commencing 24 h after the plants were placed in the field. On each day, prior to commencing the observations, we determined the total number of flowers on each plant to calculate visit frequency. We conducted a total of 3–6 observation rounds per plant pair, depending on prevailing weather conditions. Each observation round lasted for 15 min and occurred between 8:00 and 13:00 (visitation activity before 8:00 was generally very low). We alternated between the plants of each treatment, ensuring an equal number of observations of both treatments within each plot. We categorized flower visitors into three groups: honeybees, wild bees, and non-bee pollinators. We recorded a visit only when a visitor landed on a flower and engaged in nectar and/or pollen collection, excluding brief and functionally negligible visits during which visitors had minimal contact with anthers and/or stigmata. At the end of the observation days, we used organza bags to cover 10 fresh flowers, individually, on each plant and removed the remaining flowers to standardize the fruit load per plant. Before transferring the plants to a net-house on the fifth day, we enveloped each plant with cellophane paper to prevent pollen contamination during transport. Subsequently, we monitored the plants in the net-house to track pod development and recorded the number of fully developed seeds.

(c) Biological control services

To estimate biological control services received by the phytometer plants, we evaluated the following:

(1) Aphid predation rate—calculated per phytometer plant by the ratio $\frac{A_{c}-A_{o}}{A_{c}}$ where $A_{c}$ and $A_{o}$ are the numbers of aphids in the caged and uncaged treatments respectively, on day 5 in the field. This ratio serves as an index ranging from 0 to 1, with higher values corresponding to higher predation rates. On the fifth day in the field, all aphids and large nymphs (stages 3 and 4) were systematically and thoroughly counted on plant leaves and stems in both treatments. Subsequently, each plant was enveloped with a dense organza bag and transported to the laboratory for aphid parasitism determination.
(2) Aphid parasitism rate—at the end of the fifth day in the field, each infested phytometer was enclosed within an organza bag and transferred to the laboratory for the monitoring of aphid mummy development (i.e., the formation of parasitoid pupae within its host aphid) using the following method: two aphid-infested leaves per plant (50–80 adult aphids per leaf) were detached, and the petiole was inserted in water-filled tubes and placed in a monitoring cage (Figure S2). Adequate fresh leaves and water were supplied as needed for maintaining the aphids. On days 7 and 10, the number of formed mummies was recorded. The same procedure was carried out for control plants in the caged infested treatment. All emerging parasitoids were identified to the genus or species level.

### 2.5. Statistical Analyses

Data were pooled for each phytometer pair. Predation data for one plot (TZ2) were lost due to a technical problem. We used linear mixed models (lmer) and generalized linear mixed models (GLMM) to determine the effects of landscape and local habitat characteristics on the following response variables: (a) Pollinator activity—visits to flowers of infested and un-infested phytometers—overall activity, honeybees, wild bees, and non-bees. (b) Aphid predation rate on infested phytometers. (c) Aphid parasitism rate on infested photometers.

The full model for each response variable included the fixed effects of local (plot) flower abundance, local (plot) flower species richness, and the proportion of natural/semi-natural habitat within 250, 500, 750, 1000, 1500, and 2000 m radii around phytometers; a separate model was constructed for each radius, except for the 100 m radius that exhibited a weak land-use gradient (Figure S1). To test the interactive effects between pollinators and natural enemies, we added pollinator visit frequency or aphid predation and parasitism rates as fixed effects to the natural enemies and pollinator models, respectively. In addition, the date of data collection and sub-region (3 categories) were defined as random effects (see Table 1 for a summary of the full models). Pollinator activity models included two sets of analyses; one that included aphid-infested phytometers only, and the second that included both infested and un-infested phytometers with an aphid treatment variable (infested or un-infested plants) and its interaction with the landscape factor and date nested in the plot name as a random effect. In one plot (GAL), we recorded a notably high visitation frequency (though the numbers of visits and flowers were within the range of that of the other plots). Therefore, in order to avoid the overestimation of model prediction, we excluded this site from the analysis. The general trends were not affected by this exclusion (see Supplementary Results). In all pollinator visitation models, an offset variable of the number of flowers per observation was added. We utilized a Poisson distribution with a log-link function for the pollinator activity models, a binomial distribution for the parasitism rate models, and an lmer function for the log-transformed predation rate.

To determine the combined and interactive effects of pollinators and natural enemies on seed set, we utilized a GLMM with a Poisson distribution with a log-link function. The full model included the seed set of infested plants as a response variable and the pollinator visit frequency, predation rate, parasitism rate, and the interaction between them as fixed effects. The date of data collection and sub-region were defined as random effects. An offset variable of the number of counted pods per plot was added. In the case of overdispersion in count data models (pollinator activity and seed set), we used a negative binomial distribution. For all models, we calculated the variance inflation factors (VIFs) of the explanatory variables to assess the extent of multicollinearity between them. When the VIF exceeded five, the correlated variables were not included together in the same model. Finally, we performed a model selection procedure and compared alternative models for each response variable using Akaike Information Criteria corrected for sample size (AICc) [31]; models were considered equivalent if their AICc values were within delta 2. Pearson correlation was performed between the seed set and aphid herbivory level. Fruit set was not analyzed since most monitored flowers developed fruits (182/190). All analyses were performed using the packages lme4 [32], DHARMa [33], MuMin [34], and sjPlot [35] in R 4.2.2 (R Core Team 2022, Vienna, Austria). 

## 3. Results

### 3.1. Effects of Land Use on Pollination Services

A total of 2525 flower visits were recorded during 21 h of observations. Ninety-one percent of all recorded visitors were wild visitors, comprised of 77% wild bees, 21% flies, and 2% other non-bee visitors, mainly wasps and butterflies. Managed honeybees contributed 9% of all recorded flower visits. Analyses focused therefore on wild bees, flies, and honeybees.

**Figure 2:**
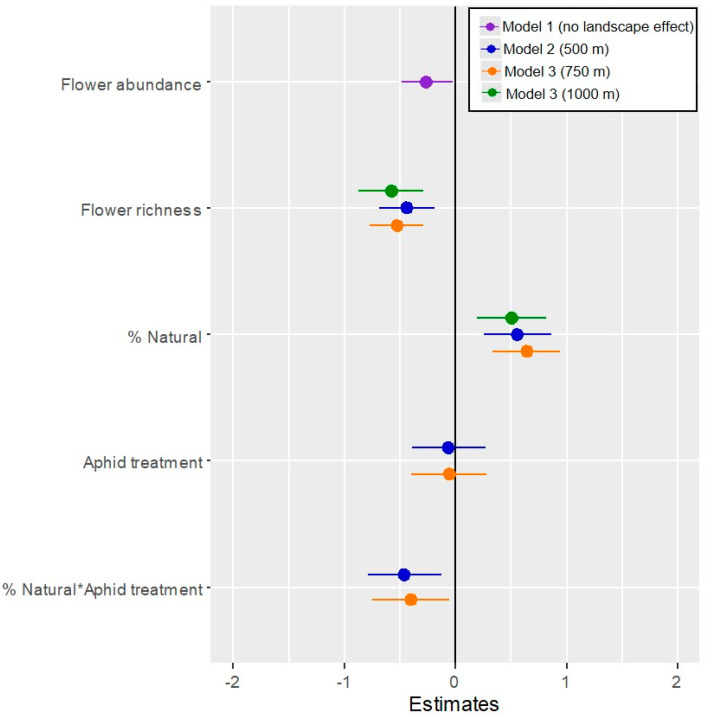
Effects of local habitat variables (flower abundance and species richness), landscape variables (% natural area at different radii around study plots), herbivory (aphid/no-aphid treatment), and the interaction between landscape variables and aphid treatment (% Natural * Aphid treatment) on overall pollinator activity according to variable estimates of the equivalent generalized linear mixed models 1–4 (delta AICc < 2).

**Figure 3:**
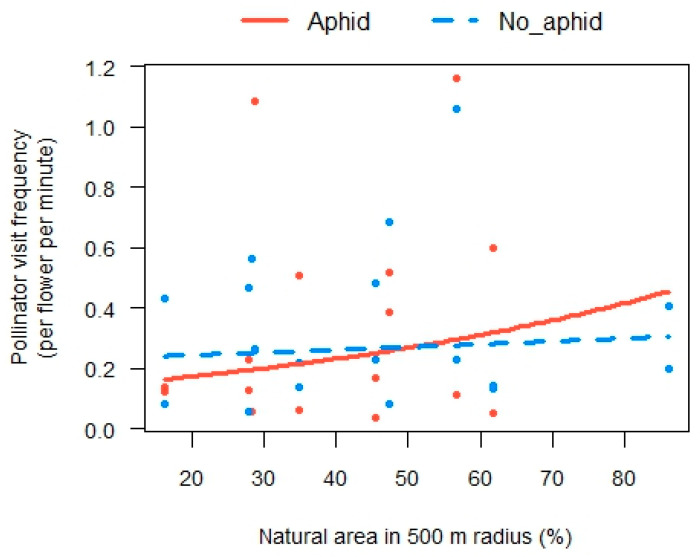
Partial residual plot showing the effect of natural/semi-natural area at a 500 m radius on pollinator activity on un-infested (blue dots) and infested (red dots) phytometers. The blue curve represents the prediction for un-infested phytometers, and the red curve represents the prediction for infested phytometers.

In general, a significant effect of the natural/semi-natural area on pollinator activity on phytometers was found within mid-range radii only (500–1000 m). The best models for overall pollinator visitation (Figure 2) described an interaction between the herbivory treatment and the natural/semi-natural area within 500 and 750 m of the phytometers, while the positive effect of the natural area was stronger for aphid-infested plants than un-infested plants (χ^2_1 d.f._^ = 7.7, *p* = 0.005 and χ ^2_1 d.f._^ = 5.1, *p* = 0.027, respectively; Figure 3 shows the model prediction for the 500 m radius, and similar predicted values were obtained for the 750 m radius). However, within a 1000 m radius, the best model predicted a positive relationship between overall pollinator activity and the natural area, without significant interaction with herbivory treatment (χ^2_1 d.f._^ = 10.4, *p* = 0.001), but with a negative relationship with local flower abundance (χ^2_1 d.f._^ = 4.9, *p* = 0.027). The best models also included the effect of habitat flower species richness and revealed a negative effect on pollinator activity (χ^2_1 d.f._^ = 11–18, *p* < 0.001, Figure 2). The natural/semi-natural area had no significant effect on pollinator activity within 250, 1500, and 2000 m. When we tested each pollinator guild separately, we found a similar interactive effect of herbivory treatment and the natural/semi-natural area on wild bee activity within the same landscape scales (500 and 750 m, χ^2_1 d.f._^ = 14.7, *p* < 0.001 and χ^2_1 d.f._^ = 9.4, *p* < 0.005, respectively) and a negative relationship with the habitat flower species richness (χ^2_1 d.f._^ = 9.7, *p* = 0.002 and χ^2_1 d.f._^ = 13.1, *p* < 0.001, respectively) (Figure S3). The natural/semi-natural area had no effect on wild bee activity within radii of 250, 1000, 1500, and 2000 m. Honeybee and fly activity did not respond to the tested explanatory variables; their overall visit frequency was very low compared to that of wild bees.

**Figure 4:**
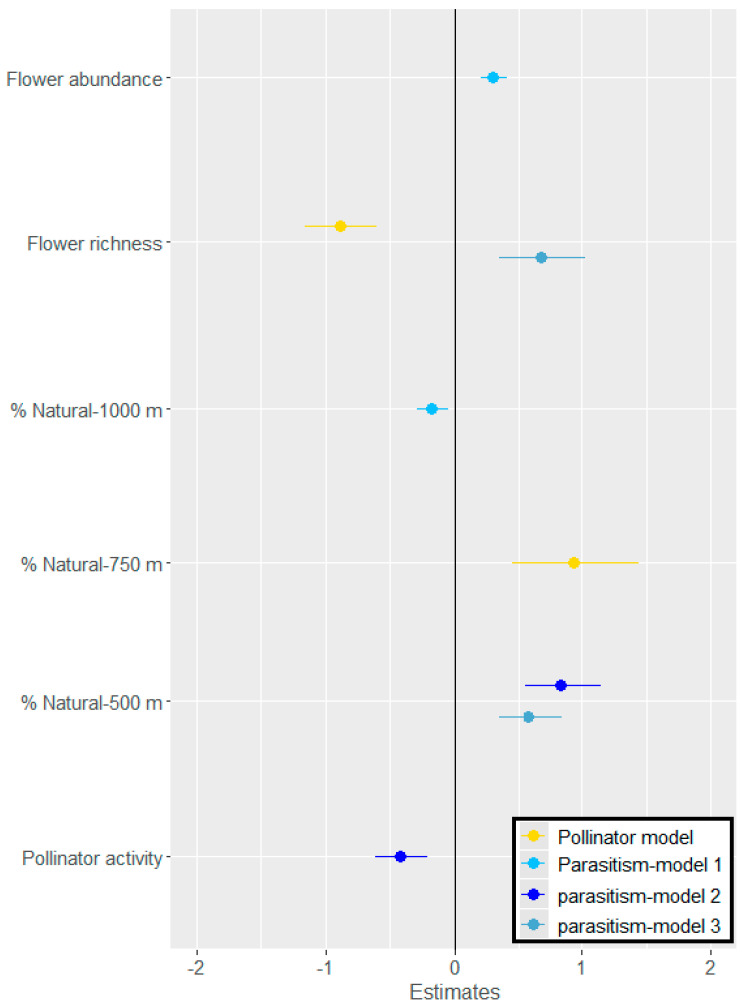
Responses of pollinator activity (yellow) and parasitism rate (shades of blue) on aphid-infested phytometers to local habitat variables (flower species richness and abundance), landscape variables (% natural area at different radii around study plots; pollinator model—750 m, Parasitism model 1—1000 m, Parasitism models 2, 3—500 m), and pollinator activity (for parasitism only). Shown are the variable estimates of the equivalent generalized linear mixed models (delta AICc < 2).

When only aphid-infested phytometers were considered (to account for possible interactions between aphids and pollinators), the overall pollinator visitation responded significantly to the natural/semi-natural area within a 750 m radius only (χ^2_1 d.f._^ = 13.6, *p* < 0.001) (Figure 4). Additionally, the model indicated a negative relationship between pollinator visitation and local flower richness (χ^2_1 d.f._^ = 38.8, *p* < 0.001). Pollinator activity did not respond significantly to aphid predation and parasitism rates.

### 3.2. Effects of Land Use on Aphid Parasitism and Predation Rates 

The following parasitoid species emerged from the experimental, aphid-infested phytometers: *Aphidius colemani*, *A. matricariae*, *Diaeretiella rapae,* and *Ephedrus* sp. (Hymenoptera: Aphidiidae). Arthropod predators were not surveyed, but *Coccinella septempunctata* (Coleoptera: Coccinellidae) adults and larvae were observed frequently on phytometers. Across study plots, the aphid parasitism rate ranged from 10 to 65 percent and the aphid predation rate varied between 10 and 90 percent. However, the predation rate did not respond significantly to the tested explanatory variables at any of the tested landscape scales. Aphid parasitism was affected negatively by the percentage of the natural area within a 1000 m radius (Figure 4, χ ^2_1 d.f._^ = 18.6, *p* < 0.001) but positively within a 500 m radius of the phytometers (Figure 4, χ ^2_1 d.f._^ = 21.3, *p* < 0.001). At this scale, a negative relationship with pollinator activity was found (χ ^2_1 d.f._^ = 19.6, *p* < 0.001). In addition, the best models described a positive relationship between the aphid parasitism rate and local flower abundance (Figure 4, χ ^2_1 d.f._^ = 19.4, *p* < 0.001) or local flower species richness (Figure 4, χ ^2_1 d.f._^ = 20.1, *p* < 0.001). 

### 3.3. Combined Effects of Natural Enemies and Pollinators on the Seed Set of Aphid-Infested Phytometers

**Figure 5:**
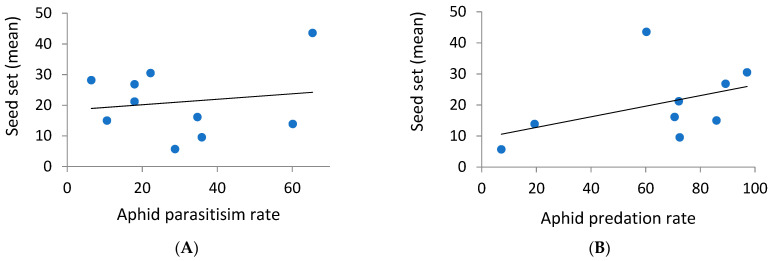
The effects of the aphid parasitism rate (**A**) and aphid predation rate (**B**) on the seed set of infested phytometers. Blue dots are observed data and lines are linear regressions.

The likelihood ratio test indicated that the parasitism and predation rates had positive effects on seed set (χ ^2_1 d.f._^ = 3.8, *p* = 0.05 and χ ^2_1 d.f._^ = 8.2, *p* = 0.004 for parasitism and predation rates, respectively) (Figure 5), although no significant effects of these biological control services were detected by a model selection procedure. Additionally, the final aphid density on experimental phytometers, as recorded on the fifth day in the field, was marginally negatively correlated with seed set (r = −0.41, *p*-value = 0.11). Finally, pollinator activity did not significantly affect the seed set of aphid-infested phytometers.

## 4. Discussion

The aim of the present study was to investigate the combined and interactive effects of land-use characteristics on the concurrent provision of pollination and biological pest control services and ultimately on plant seed set. Our findings revealed that pollinators and natural enemies responded differently to land-use characteristics; their responses were scale-dependent, and the activity of pollinators was interactively affected by aphid infestation and landscape characteristics. Overall, we observed a positive effect of aphid predation and parasitism rates on the seed production of aphid-infested phytometers, but no inter-guild interactive effects were detected between pollinators and natural enemies. As this is one of the very few studies that examined the combined and interactive activity of several guilds of ecosystem service providers across a land-use gradient in an agro-ecosystem, it sets the basis for a broader, cross-guild perspective of studying and managing ecosystem service providers in agro-ecosystems. 

### 4.1. Pollinator and Natural Enemy Responses to Landscape and Habitat Characteristics 

First, the two guilds of natural enemies exhibited different responses to landscape and habitat variables in our study; parasitism responded to all predictors whereas predation did not display any such response to the same set of variables. These differential responses could be attributed to differences in biology, foraging strategies, diet, and other life history traits between parasitoid wasps and predatory arthropods. For example, differences between parasitoid and predator responses were found in movement behavior within agro-ecosystems; whereas aphid parasitoids commonly move between native vegetation and adjacent crop plants, and predators move less frequently between these habitats [36]. Predators are known to respond more strongly to other factors, such as the vegetation structure, ephemerality, distance to hedgerows, and the presence of other prey [37], which were not investigated in the present study. Pollinator guilds also differed in their responses to the tested variables; while wild bees, the majority of recorded visitors to phytometers, were affected by landscape and local habitat characteristics, as well as their interaction with herbivory, honeybees and flies did not exhibit such responses. This may stem from various biological and ecological differences between these guilds, in addition to a low statistical power because of low counts of honeybees and flies.

The response of parasitoids and pollinators to land use differed across landscape scales; whereas pollinator activity responded positively to the proportion of the natural/semi-natural area within a 500–1000 m radius of the phytometers, the parasitism rate switched from a positive response at short distances (500 m radius) to a negative one at the large scale (1000 m radius). The positive effect of natural/semi-natural areas on pollinators, natural enemies, and their respective ecosystem services has been well documented in other systems [2,21,38,39]. In our study, land-use at the mid-range radius was particularly relevant, possibly reflecting the relative short dispersal range of parasitoids and small wild bees, the main pollinator group in our system [27,30]. This positive effect of the natural/semi natural habitat area at the short–intermediate range on the aphid parasitism rate may be attributed to nectar and pollen provisioning by flowering natural vegetation. Such resources are essential for parasitoid fecundity, flight, and longevity [40]. However, our results indicate that this parasitoid facilitation was lost at a larger spatial scale; a negative effect of natural/semi-natural habitats on aphid parasitism was detected at the 1000 m radius. Such negative effects may occur when croplands provide more important resources for natural enemies than do natural habitats [41]. Because all of the main parasitoid species identified in our study attack a wide range of aphid hosts, the negative effect of natural/semi-natural habitats at the 1000 m radius on aphid parasitism may be attributed to the availability of alternative hosts on crop plants nearby, as well as to their limited dispersal ability. It is important to acknowledge, however, that the classification of natural/semi-natural areas as a single variable may have oversimplified the complexity within this category; habitat types, vegetation traits, and the quantity and quality of foraging and nesting resources vary considerably within these two land-use categories [42,43]. Therefore, future research should consider these factors, for example, by the use of a resource landscape approach [44]. Such an approach promises to yield a more comprehensive understanding of the influence of natural/semi-natural areas on guild communities and the ecosystem services they provide. 

Our results reveal contrasting effects of local flower abundance and species richness on the aphid parasitism rate and pollinator activity. We detected a positive relationship between the aphid parasitism rate and flower abundance and richness, indicating that higher levels of floral resources contribute to the provision of pest control services by providing parasitoids with needed food sources and supporting higher wasp populations. This finding is in agreement with the ‘parasitoid nectar provision hypothesis’ [40], which suggests that providing nectar resources for parasitoids can enhance pest control in agricultural fields. However, it is worth noting that several of the studies that tested this hypothesis directly did not find any improvement in pest control when nectar resources were added in the field [45]. On the other hand, we found a negative relationship between pollinator activity and flower abundance and species richness nearby. This result may be indicative of a competitive relationship between our phytometers and native plants in flower-abundant and species-rich habitats. If so, it appears that our phytometers may be less attractive to pollinators than wild plants in such environments, as was demonstrated in various systems with co-blooming plants [46,47,48,49]. These contrasting responses of various insect guilds to habitat flower resources suggest that different mechanisms govern these effects and, in turn, the provided ecosystem services.

### 4.2. Interactions among Pollinators, Herbivores, and the Pollination and Pest Control Services They Deliver across Landscape Gradients

The effect of herbivory on pollination services was recorded by two key parameters: pollinator activity and seed set. The results showed a marginal yet clear decline in phytometer seed set with increasing levels of herbivory, likely due to the stress caused, which in turn impairs phytometers’ productivity [50]. However, the presence of aphids on phytometer plants interacted with the effect of land-use on pollination services; the positive effect of natural/semi-natural surroundings on pollinator activity was more pronounced on infested than un-infested phytometer plants. This interaction effect between herbivory and land-use may reflect changes in the flower characteristics of aphid-infested plants that affect their attractiveness to pollinators. These results are in line with the well-established effect of herbivory on various floral traits, such as flower number and nectar and pollen production [51,52,53,54,55,56] and the subsequent reduction in plant attractiveness to pollinators [57]. The competitive interactions between phytometers and surrounding flowers are also supported by the negative effects found for local flower species richness and abundance in the study plots on pollinator activity on the phytometers. Overall, our findings highlight substantial direct and indirect, through the plant, relationships between pollinators and herbivores and their interactive responses to landscape properties. 

Finally, we found a negative effect of pollinator activity on the parasitism rate, suggesting a competitive relationship between these two guilds. These results may be due to the direct interference of bees to parasitoid access to phytometer flowers or may reflect an indirect, plant-mediated competitive depletion of flower nectar resources shared by the adults of the two guilds. These results are in agreement with a few earlier studies that also reported such competitive relationships between parasitoid wasps and pollinators [14,58]. 

### 4.3. Combined Effects of Natural Enemies and Pollinators on Seed Set by Aphid-Infested Phytometers

We did not find a clear relationship between pollinator activity and seed set in our study system. It is plausible that in this particular system, pollination services did not act as a limiting factor for plant reproductive success. Alternatively, other factors that influence seed setting may have obscured any contribution by the pollinators. For example, aphid parasitism and predation rates contributed to seed production by aphid-infested phytometers whereas aphid herbivory had a negative, though not significant, effect on seed set. Further research with larger sample sizes is needed to confirm these trends. It is important to acknowledge, however, that our findings are based on model plants (i.e., phytometers). To provide more mechanistic understanding of these interactions, further research should be conducted in more realistic cropping systems.

It Is important to note, however, that parasitoids and predators are expected to respond positively to aphid infestation. Enemies are known to aggregate on host/prey patches, and many species remain on such plants to feed on aphid-produced honeydew. Therefore, any two-way interaction among aphids, pollinators, and natural enemies is likely to be influenced by the third guild in the system. These complex interactions are then influenced differentially by resource availability in various habitats and distances in the landscape, a major challenge to tease apart and study empirically. 

## 5. Conclusions

Our study has revealed the pivotal role that natural and semi-natural habitats may play in mitigating the adverse effects of aphid infestation on pollination services. Additionally, we found that different groups of ecosystem service providers may respond differently to landscape and local habitat factors. These idiosyncratic responses, observed in both pollinators and natural enemies, may stem from their unique biology and behavior, distinct relationships with local or landscape foraging resources, or interactive effects. Therefore, it is important to gain a more mechanistic understanding of these complex interactions, as they may have significant implications for management recommendations aimed at improving the provision of pollination and pest control services and ultimately increasing crop yields in agro-ecosystems. Such research findings could allow the development of sustainable, low-input Integrated Pest and Pollinator Management (IPPM) systems.

## Figures and Tables

**Table 1:** Summary of the full models’ structure: response variables, model type, predictors, and fixed and random effects.

Response Variable (& Model Type)	Fixed Effects Predictors	Random Effects
Number of visits in flowers - Infested & un-infested phytometers: Overall pollinators Honeybees Wild bees Flies (Glmer, Poisson/ glmer, Negative binomial)	Proportion of natural/semi-natural area within: 250–2000 m radius (1) Aphid treatment Aphid treatment * % natural/semi-natural Local flower abundance Local flower species richness Number of flowers [offset variable]	Date/plot (2) Sub-region
Number of visits in flowers - Infested phytometers only: Overall pollinators Honeybees Wild bees Flies (Glmer, Poisson/ glmer, negative binomial)	Proportion of natural/semi-natural area within: 250–2000 m radius (1) Aphid treatment Aphid treatment * % natural/semi-natural Local flower abundance Local flower species richness Aphid predation rate Aphid parasitism rate Number of flowers [offset variable]	Date Sub-region
Aphid predation rate (lmer) Aphid parasitism rate (glmer, binomial)	Proportion of natural/semi-natural area within: 250–2000 m radius (1) Local flower abundance Local flower species richness Pollinator visit frequency	Date Sub-region
Seed set in infested phytometers (Glmer, Poisson/ glmer, Negative binomial)	Pollinator visit frequency * Aphid predation rate Pollinator visit frequency * Aphid parasitism rate Aphid predation rate * Aphid parasitism rate Number of pods [offset variable]	Date Sub-region
(1) A separated model for each radius		
(2) Date nested in plot * for interaction		

## Data Availability

Data are contained in Table S1.

## Supplementary Materials

The following supporting information can be downloaded at: https://www.mdpi.com/article/10.3390/insects14110872/s1, Figure S1: Proportions of the natural/semi-natural area within a radius of 100–2000 m around the study plots. Figure S2: Monitoring the development of aphid mummies: (A) Monitoring cages, (B) Leaf on the first day, (C) Mummies on the 7th day. Figure S3: Generalized linear mixed best model estimates of the effects of landscape, local flower species richness, and herbivory on wild bee activity (similar results for wild bee activity within 750 m). Supplementary Results: Results of the pollinator activity models of aphid-infested and un-infested phytometers. Dataset S1: Data summarized by treatment and plot analyzed in the study.

## References

[1] Power A.G. (2010). Ecosystem services and agriculture: Tradeoffs and synergies. Philos. Trans. R. Soc. B Biol. Sci..

[2] Dainese M., Martin E.A., Aizen M.A., Albrecht M., Bartomeus I., Bommarco R., Carvalheiro L.G., Chaplin-Kramer R., Gagic V., Garibaldi L.A. (2019). A global synthesis reveals biodiversity-mediated benefits for crop production. Sci. Adv..

[3] Klein A.-M., Vaissiere B.E., Cane J.H., Steffan-Dewenter I., Cunningham S.A., Kremen C., Tscharntke T. (2007). Importance of pollinators in changing landscapes for world crops. Proc. R. Soc. B Biol. Sci..

[4] Losey J.E., Vaughan M. (2006). The economic value of ecological services provided by insects. BioScience.

[5] Bommarco R., Kleijn D., Potts S.G. (2013). Ecological intensification: Harnessing ecosystem services for food security. Trends Ecol. Evol..

[6] Jeanneret P., Aviron S., Alignier A., Lavigne C., Helfenstein J., Herzog F., Kay S., Petit S. (2021). Agroecology landscapes. Landsc. Ecol..

[7] Lundin O., Rundlöf M., Jonsson M., Bommarco R., Williams N.M. (2021). Integrated pest and pollinator management—Expanding the concept. Front. Ecol. Environ..

[8] Jeavons E., Le Lann C., van Baaren J. (2023). Interactions between natural enemies and pollinators: Combining ecological theory with agroecological management. Entomol. Gen..

[9] Garibaldi L.A., Andersson G.K.S., Requier F., Fijen T.P.M., Hipólito J., Kleijn D., Pérez-Méndez N., Rollin O. (2018). Complementarity and synergisms among ecosystem services supporting crop yield. Glob. Food Secur..

[10] Lundin O., Smith H.G., Rundlof M., Bommarco R. (2013). When ecosystem services interact: Crop pollination benefits depend on the level of pest control. Proc. R. Soc. B Biol. Sci..

[11] Sutter L., Albrecht M. (2016). Synergistic interactions of ecosystem services: Florivorous pest control boosts crop yield increase through insect pollination. Proceedings. Biol. Sci. R. Soc..

[12] Albrecht M., Schmid B., Hautier Y., Mueller C.B. (2012). Diverse pollinator communities enhance plant reproductive success. Proc. R. Soc. B Biol. Sci..

[13] Lindstrom S.A.M., Herbertsson L., Rundlof M., Bommarco R., Smith H.G. (2016). Experimental evidence that honeybees depress wild insect densities in a flowering crop. Proc. R. Soc. B Biol. Sci..

[14] Campbell A.J., Biesmeijer J.C., Varma V., Wäckers F.L. (2012). Realising multiple ecosystem services based on the response of three beneficial insect groups to floral traits and trait diversity. Basic Appl. Ecol..

[15] Jeavons E., van Baaren J., Le Lann C. (2020). Resource partitioning among a pollinator guild: A case study of monospecific flower crops under high honeybee pressure. Acta Oecologica.

[16] Greco C.F., Kevan P.G. (1995). Patch choice in the anthophilous ambush predator Phymata americana: Improvement by switching hunting sites as part of the initial choice. Can. J. Zool. Rev. Can. Zool..

[17] Sinu P.A., Sibisha V.C., Reshmi M.V.N., Reshmi K.S., Jasna T.V., Aswathi K., Megha P.P. (2017). Invasive ant (Anoplolepis gracilipes) disrupts pollination in pumpkin. Biol. Invasions.

[18] Gagic V., Marcora A., Howie L. (2019). Additive and interactive effects of pollination and biological pest control on crop yield. J. Appl. Ecol..

[19] Shackelford G., Steward P.R., Benton T.G., Kunin W.E., Potts S.G., Biesmeijer J.C., Sait S.M. (2013). Comparison of pollinators and natural enemies: A meta-analysis of landscape and local effects on abundance and richness in crops. Biol. Rev..

[20] Veres A., Petit S., Conord C., Lavigne C. (2013). Does landscape composition affect pest abundance and their control by natural enemies? A review. Agric. Ecosyst. Environ..

[21] Holland J.M., Douma J.C., Crowley L., James L., Kor L., Stevenson D.R.W., Smith B.M. (2017). Semi-natural habitats support biological control, pollination and soil conservation in Europe. A review. Agron. Sustain. Dev..

[22] Karp D.S., Chaplin-Kramer R., Meehan T.D., Martin E.A., DeClerck F., Grab H., Gratton C., Hunt L., Larsen A.E., Martinez-Salinas A. (2018). Crop pests and predators exhibit inconsistent responses to surrounding landscape composition. Proc. Natl. Acad. Sci. USA.

[23] Bartual A.M., Sutter L., Bocci G., Moonen A.C., Cresswell J., Entling M., Giffard B., Jacot K., Jeanneret P., Holland J. (2019). The potential of different semi-natural habitats to sustain pollinators and natural enemies in European agricultural landscapes. Agric. Ecosyst. Environ..

[24] Albrecht M., Kleijn D., Williams N.M., Tschumi M., Blaauw B.R., Bommarco R., Campbell A.J., Dainese M., Drummond F.A., Entling M.H. (2020). The effectiveness of flower strips and hedgerows on pest control, pollination services and crop yield: A quantitative synthesis. Ecol. Lett..

[25] Martin E.A., Dainese M., Clough Y., Baldi A., Bommarco R., Gagic V., Garratt M.P.D., Holzschuh A., Kleijn D., Kovacs-Hostyanszki A. (2019). The interplay of landscape composition and configuration: New pathways to manage functional biodiversity and agroecosystem services across Europe. Ecol. Lett..

[26] Egan P.A., Dicks L.V., Hokkanen H.M.T., Stenberg J.A. (2020). Delivering Integrated Pest and Pollinator Management (IPPM). Trends Plant Sci..

[27] Pisanty G., Mandelik Y. (2015). Profiling crop pollinators: Life history traits predict habitat use and crop visitation by Mediterranean wild bees. Ecol. Appl..

[28] Weizel Y., Polak G., Cohen Y. (1978). Ecology of the Vegetation in Israel.

[29] Blackman R.L., Eastop V.F. (2000). Aphids on the World’s Crops: An Identification and Information Guide.

[30] Roth T., Coll M., Mandelik Y. (2023). The role of uncultivated habitats in supporting wild bee communities in Mediterranean agricultural landscapes. Diversity.

[31] Johnson J.B., Omland K.S. (2004). Model selection in ecology and evolution. Trends Ecol. Evol..

[32] Bates D., Mächler M., Bolker B., Walker S. (2015). Fitting Linear Mixed-Effects Models Using lme4. J. Stat. Softw..

[33] Hartig F. (2022). DHARMa: Residual Diagnostics for Hierarchical (Multi-Level/Mixed) Regression Models. R Package Version 0.4.6.

[34] Bartoń K. (2023). MuMIn:Multi-Model Inference. R Package Version 1.47.5.

[35] Lüdecke D. (2023). sjPlot:Data Visualization for Statistics in Social. R Package Version 2.8.14.

[36] Macfadyen S., Muller W. (2013). Edges in agricultural landscapes: Species interactions and movement of natural enemies. PLoS ONE.

[37] Gurr G.M., Wratten S.D., Landis D.A., You M.S. (2017). Habitat Management to Suppress Pest Populations: Progress and Prospects. Annu. Rev. Entomol..

[38] Kennedy C.M., Lonsdorf E., Neel M.C., Williams N.M., Ricketts T.H., Winfree R., Bommarco R., Brittain C., Burley A.L., Cariveau D. (2013). A global quantitative synthesis of local and landscape effects on wild bee pollinators in agroecosystems. Ecol. Lett..

[39] Steffan-Dewenter I., Munzenberg U., Burger C., Thies C., Tscharntke T. (2002). Scale-dependent effects of landscape context on three pollinator guilds. Ecology.

[40] Heimpel G.E., Jervis M.A. (2005). Does floral nectar improve biological control by parasitoids. Plant-Provided Food for Carnivorous Insects: A Protective Mutualism and Its Applications.

[41] Tscharntke T., Karp D.S., Chaplin-Kramer R., Batary P., DeClerck F., Gratton C., Hunt L., Ives A., Jonsson M., Larsen A. (2016). When natural habitat fails to enhance biological pest control—Five hypotheses. Biol. Conserv..

[42] Duflot R., Aviron S., Ernoult A., Fahrig L., Burel F. (2015). Reconsidering the role of ‘semi-natural habitat’ in agricultural landscape biodiversity: A case study. Ecol. Res..

[43] Rosas-Ramos N., Banos-Picon L., Tormos J., Asis J.D. (2020). Natural enemies and pollinators in traditional cherry orchards: Functionally important taxa respond differently to farming system. Agric. Ecosyst. Environ..

[44] Nottebrock H., Schmid B., Mayer K., Devaux C., Esler K.J., Bohning-Gaese K., Schleuning M., Pagel J., Schurr F.M. (2017). Sugar landscapes and pollinator-mediated interactions in plant communities. Ecography.

[45] Heimpel G.E. (2019). Linking parasitoid nectar feeding and dispersal in conservation biological control. Biol. Control.

[46] Holzschuh A., Dainese M., Gonzalez-Varo J.P., Mudri-Stojnic S., Riedinger V., Rundlof M., Scheper J., Wickens J.B., Wickens V.J., Bommarco R. (2016). Mass-flowering crops dilute pollinator abundance in agricultural landscapes across Europe. Ecol. Lett..

[47] Evans T.M., Cavers S., Ennos R., Vanbergen A.J., Heard M.S. (2017). Florally rich habitats reduce insect pollination and the reproductive success of isolated plants. Ecol. Evol..

[48] Grab H., Blitzer E.J., Danforth B., Loeb G., Poveda K. (2017). Temporally dependent pollinator competition and facilitation with mass flowering crops affects yield in co-blooming crops. Sci. Rep..

[49] Xie Z.H., Wang J.M., Pan D.D., An J.D. (2019). Landscape-modified concentration effect and waylaying effect of bees and their consequences on pollination of mass-flowering plants in agricultural ecosystems. Agric. Ecosyst. Environ..

[50] Oerke E.C. (2006). Crop losses to pests. J. Agric. Sci..

[51] Rusman Q., Poelman E.H., Nowrin F., Polder G., Lucas-Barbosa D. (2019). Floral plasticity: Herbivore-species-specific-induced changes in flower traits with contrasting effects on pollinator visitation. Plant Cell Environ..

[52] Strauss S.Y., Conner J.K., Rush S.L. (1996). Foliar herbivory affects floral characters and plant attractiveness to pollinators: Implications for male and female plant fitness. Am. Nat..

[53] Lehtila K., Strauss S.Y. (1997). Leaf damage by herbivores affects attractiveness to pollinators in wild radish, *Raphanus raphanistrum*. Oecologia.

[54] Krupnick G.A., Weis A.E., Campbell D.R. (1999). The consequences of floral herbivory for pollinator service to Isomeris arborea. Ecology.

[55] Mothershead K., Marquis R.J. (2000). Fitness impacts of herbivory through indirect effects on plant-pollinator interactions in Oenothera macrocarpa. Ecology.

[56] Barber N.A., Adler L.S., Theis N., Hazzard R.V., Kiers E.T. (2012). Herbivory reduces plant interactions with above- and belowground antagonists and mutualists. Ecology.

[57] Moreira X., Castagneyrol B., Abdala-Roberts L., Traveset A. (2019). A meta-analysis of herbivore effects on plant attractiveness to pollinators. Ecology.

[58] Lee J., Heimpel G. (2003). Nectar availability and parasitoid sugar feeding. Proceedings of the 1st International Symposium on Biological Control of Arthropods.

