# Supplementary material for: Complex Effects of a Land-Use Gradient on Pollinators and Natural Enemies: Natural Habitats Mitigate the Effects of Aphid Infestation on Pollination Services
Source: Insects. 2023 Nov 13;14(11):872. doi: 10.3390/insects14110872 (PMC10672408; doi:10.3390/insects14110872)
Supplement: Supplementary file 1 [file insects-14-00872-s001.zip › Combined pollinator herbivores effects_7_11_23_SUPPLEMENTARY MATERIAL AS SUBMITTED.pdf]

Figure S1: Proportions of natural/semi-natural area within radius of 100-2000 m around study plots.

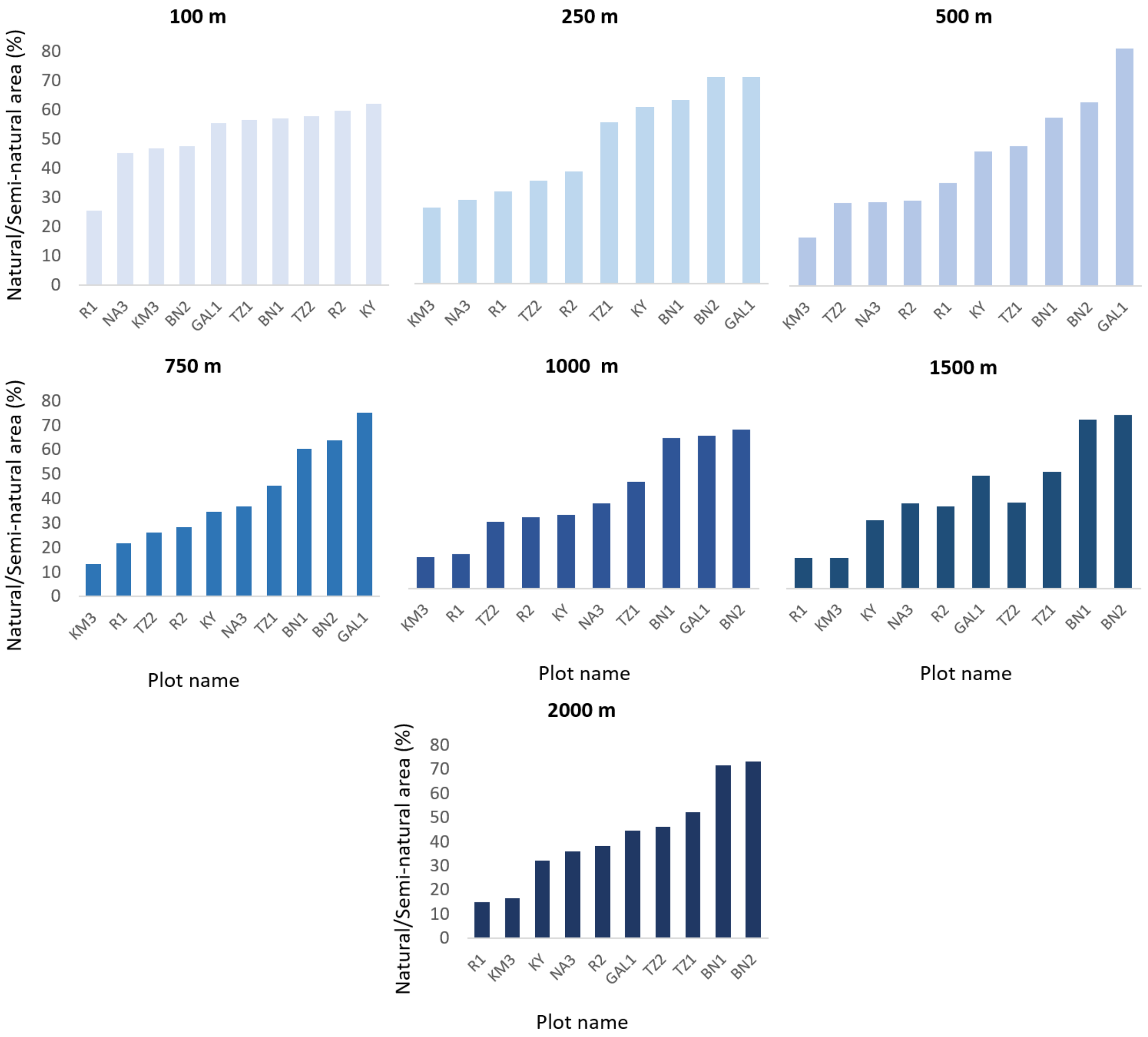

Figure S2: Monitoring the development of aphid mummies: A) Monitoring cages, B) Leaf on the first day, C) Mummies on the 7th day.

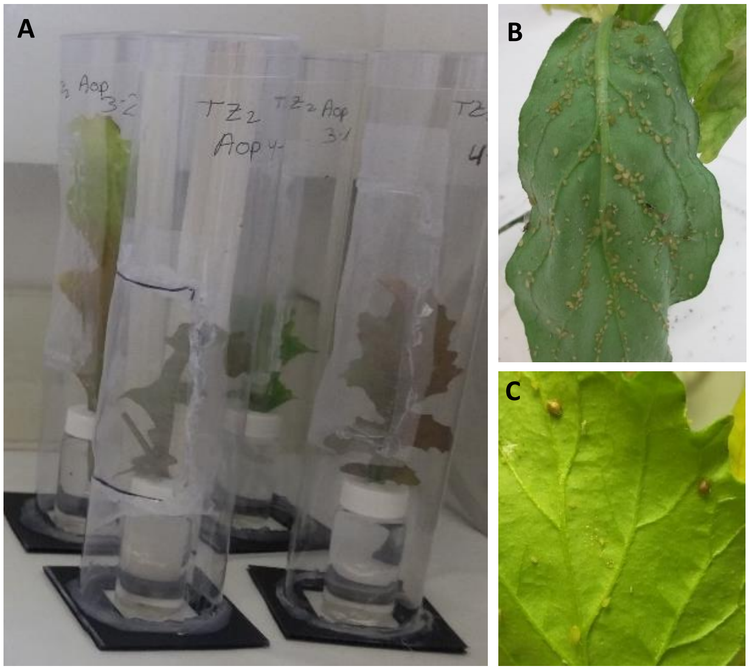

Figure S3: Generalized linear mixed best model estimates of the effects of landscape, local flower species richness and herbivory on wild bee activity (similar results for wild bee activity within 750 m).

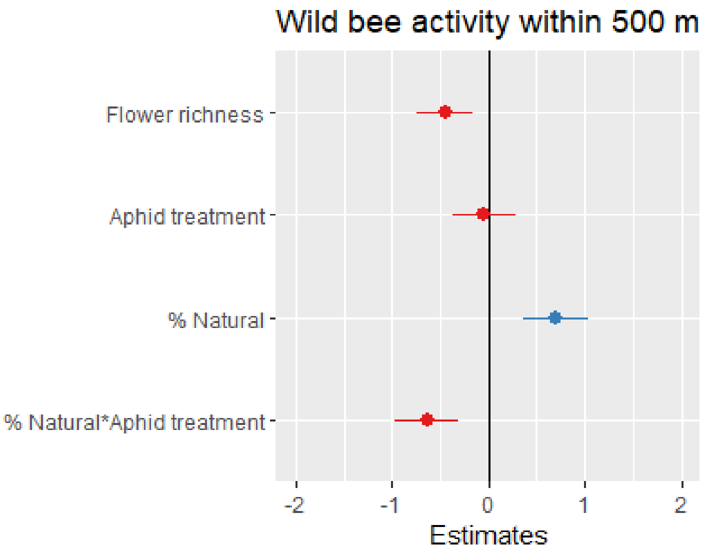

Supplementary Results: Results of pollinator activity models of aphid infested and un-infested phytometers: included the GAL plot: The best models for overall pollinator visitation describe an interaction between herbivory treatment and natural/semi-natural area within 500 and 750 m radius of phytometers, while a positive effect of natural area is stronger for aphid-infested plants than un-infested plants ($\chi^2_{1\ d.f.}$=7.1, p=0.007 and $\chi^2_{1\ d.f.}$=4.9, p=0.026 respectively; However, within 1000 m radius, the best model predicted a positive relationship (marginal significance) between overall pollinator activity and natural area, without significant interaction with herbivory treatment ($\chi^2_{1\ d.f.}$=3.3, p=0.068), but with a negative relationship (marginal significance) with local flower abundance ($\chi^2_{1\ d.f.}$=3.3, p=0.068). Best models include also the effect of habitat flower species richness and revealed a negative effect on pollinator activity ($\chi^2_{1\ d.f.}$=11-18, p<0.001).
